# The usefulness of F-18 FDG PET/CT-mammography for preoperative staging of breast cancer: comparison with conventional PET/CT and MR-mammography

**DOI:** 10.2478/raon-2013-0031

**Published:** 2013-10-08

**Authors:** Eun-Ha Moon, Seok Tae Lim, Yeon-Hee Han, Young Jin Jeong, Yun-Hee Kang, Hwan-Jeong Jeong, Myung-Hee Sohn

**Affiliations:** 1 Department of Nuclear Medicine, Chonbuk National University Medical School and Hospital, Jeonju, Jeonbuk, Korea; 2 Research Institute of Clinical Medicine, Chonbuk National University Medical School and Hospital, Jeonju, Jeonbuk, Korea; 3 Department of Nuclear Medicine, Presbyterian Medical Center, Jeonju, Jeonbuk, Korea; 4 Department of Nuclear Medicine, Dong-A University Medical Center, Busan, Korea; 5 Department of Nuclear Medicine, Eulji University School of Medicine, Daejeon, Korea

**Keywords:** breast cancer, fluorodeoxyglucose, positron emission tomography, MRI, mammography

## Abstract

**Background:**

The objective of the study was to compare the diagnostic efficacy of an integrated Fluorine-18 fluorodeoxyglucose (F-18 FDG) PET/CT-mammography (mammo-PET/CT) with conventional torso PET/CT (supine-PET/CT) and MR-mammography for initial assessment of breast cancer patients.

**Patients and methods:**

Forty women (52.0 ± 12.0 years) with breast cancer who underwent supine-PET/CT, mammo-PET/CT, and MR-mammography from April 2009 to August 2009 were enrolled in the study. We compared the size of the tumour, tumour to chest wall distance, tumour to skin distance, volume of axillary fossa, and number of meta-static axillary lymph nodes between supine-PET/CT and mammo-PET/CT. Next, we assessed the difference of focality of primary breast tumour and tumour size in mammo-PET/CT and MR-mammography. Histopathologic findings served as the standard of reference.

**Results:**

In the comparison between supine-PET/CT and mammo-PET/CT, significant differences were found in the tumour size (supine-PET/CT: 1.3 ± 0.6 cm, mammo-PET/CT: 1.5 ± 0.6 cm, p < 0.001), tumour to thoracic wall distance (1.8 ± 0.9 cm, 2.2 ± 2.1 cm, p < 0.001), and tumour to skin distance (1.5 ± 0.8 cm, 2.1 ± 1.4 cm, p < 0.001). The volume of axillary fossa was significantly wider in mammo-PET/CT than supine-PET/CT (21.7 ± 8.7 cm^3^ *vs.* 23.4 ± 10.4 cm^3^, p = 0.03). Mammo-PET/CT provided more correct definition of the T-stage of the primary tumour than did supine-PET/CT (72.5% *vs*. 67.5%). No significant difference was found in the number of metastatic axillary lymph nodes. Compared with MR-mammography, mammo-PET/CT provided more correct classification of the focality of lesion than did MR-mammography (95% *vs*. 90%). In the T-stage, 72.5% of cases with mammo-PET/CT and 70% of cases with MR-mammography showed correspondence with pathologic results.

**Conclusions:**

Mammo-PET/CT provided more correct definition of the T-stage and evaluation of axillary fossa may also be delineated more clearly than with supine-PET/CT. The initial assessment of mammo-PET/CT would be more useful than MR-mammography because the mammo-PET/CT indicates similar accuracy with MR-mammography for decision of T-stage of primary breast tumour and more correct than MR-mammography for defining focality of lesion.

## Introduction

Breast cancer is the most common cancer and the second leading cause of cancer-related death in women in western countries.^1^ Currently, breast cancer is diagnosed by triple assessment of physical examination, radiologic evaluation, and histopathologic confirmation. In particular, radiologic evaluation is more important when the size of tumour is too small.^2^,^3^ Treatment strategy of breast cancer is dependent on the size of the breast, size or extent of the tumour, infiltration, and lymph node metastasis, et cetera. Therefore, once breast cancer is diagnosed, accurate staging of the tumour is extremely important as it influences appropriate treatment decision and determines prognosis.^4^

Initial breast cancer staging has been based on a multimodality approach: X-ray mammography, ultrasonography, MRI, sentinel lymph node biopsy, and bone scintigraphy.^5^ X-ray mammography is the most widely used technique for evaluation of the primary tumour in both symptomatic and asymptomatic patients.^6^–^8^ However, because of dense breasts, X-ray mammography is less sensitive for Korean women, and underestimates the size or focality of the primary tumour.^2^,^3^,^9^,^10^ Correlation of X-ray mammography findings with breast ultrasonography and MRI has been helpful for differential diagnosis of a primary breast lesion and for detection of occult breast tumours.^11^–^13^ However, this multimodality approach is invasive, as well as time and cost consuming. Thus, a noninvasive, single-session approach for breast cancer staging may be desirable.^14^–^16^

Fluorine-18 fluorodeoxyglucose positron emission tomography/computed tomography (F-18 FDG PET/CT) allows for accurate staging of various types of malignancies and for acquisition of whole-body imaging.^17^,^18^ Thus, PET/CT has high sensitivity in detection of primary tumour and axillary lymph node metastasis, as well as distant metastasis, in patients with breast cancer. In addition, PET/CT is beneficial for use in monitoring of therapy. However, with CT as part of PET/CT, tumour delineation is more difficult to determine than with MRI up to now, PET/CT has been used predominantly for evaluation of distant metastases.^19^–^23^ MR-mammography, as well as X-ray mammography, in conjuction with ultrasonography, has remained the method of choice for imaging of the primary tumour of the breast. Patient positioning, which is similar to that used in MR-mammography (PET/CT-mammography), may provide more accurate information on the primary tumour and axillary lymph node in PET/CT. However, the diagnostic accuracy of this PET/CT-mammography algorithm has not yet been defined.

The purpose of this study was to assess the usefulness and feasibility of PET/CT-mammography for preoperative staging of breast cancer and to compare the capability of measurement and delineation of lesion of PET/CT-mammography to those of conventional torso F-18 FDG PET/CT and MR-mammography.

## Patients and methods

### Patients

Forty women (mean age; 52.0 ± 12.0 years) with breast cancer who underwent conventional torso F-18 FDG PET/CT (supine-PET/CT), PET/CT-mammography (mammo-PET/CT) (PET/CT-mammography was acquired after the conventional torso PET/CT), and MR-mammography from April 2009 to August 2009 were enrolled (mean interval between MR-mammography and PET/CT-mammography: 1.7 ± 3.9 days, range: 0–23 days). All patients were confirmed breast cancer by biopsy before the diagnostic imagings were acquired. We compared the primary tumour size (longest diameter of lesion in trans-axial image), tumour to thoracic wall distance (T-C distance, maximal distance from tumour to thoracic wall in trans-axial image), tumour to skin distance (T-S distance, maximal distance from tumour to skin in trans-axial image), volume of ipsilateral axillary fossa, and number of suspected metastatic axillary lymph nodes between supine-PET/CT and mammo-PET/CT, retrospectively. Next, we evaluated the difference of the focality of the primary tumour and the size of the tumour in mammo-PET/CT and MR-mammography, retrospectively. Histopathologic findings served as the standard of reference. The protocol was approved by the Institutional Review Board of Chonbuk National University Hospital.

### Imaging protocol

#### Supine-PET/CT and mammo-PET/CT

**FIGURE 1.**
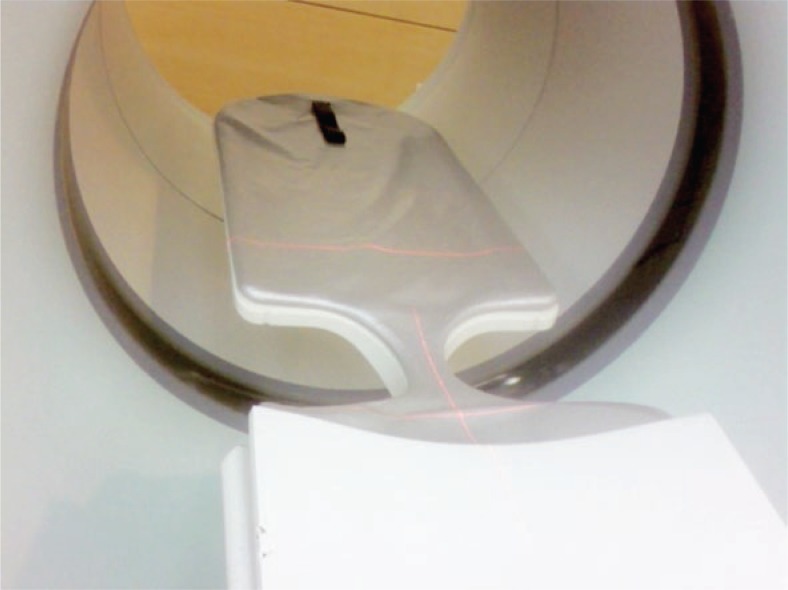
Breast positioning device. The device is constructed for prone breast positioning.

Whole body F-18 FDG PET/CT scans were obtained on a BiographTruepoint 40 PET/CT (Siemens, Berlin, Germany). Patients fasted for at least 6 hours before receiving an intravenous injection of F-18 FDG (mean: 429.0 ± 59.0 MBq (11.6 ± 1.6 mCi), range: 259.0 – 592.0 MBq (7 – 16 mCi)). Prior to injection of F-18 FDG, blood glucose levels were checked in order to determine whether the levels were within the reference range (< 150 mg/dL). PET/CT images were acquired at 50 – 60 minutes after intravenous administration of F-18 FDG. Torso PET/CT from the skull base to the upper thigh was performed while the patient was in supine position (5 bed position, 120 seconds per bed). An iodinated contrast agent containing 320 mg of iodine per millilitre was injected using an automated injector (2.0 ml/kg body weight at an injection rate of 1.6 ml/s). A limited breath-hold technique was used in order to avoid motion-induced artifacts near the diaphragm. Then, mammo-PET/CT was performed after repositioning the patient prone (2 bed position, 120 seconds per bed), using a special breast-positioning device (E-cam scintimammography pallet, Siemens) (Figure 1). That device allowed for a breast position similar to that for the MR-mammography used in a routine clinical setting. Mammo-PET/CT image acquisition started at approximately 75 minutes after intravenous injection of F-18 FDG. The PET images were reconstructed by standard 3D-iterative algorithm (ordered subset expectation maximization; OSEM).

#### MR-mammography

All patients underwent MR-mammography. Dynamic contrast-enhanced breast MRI (Magnetom Symphony 1.5T (Siemens, Berlin, Germany)) was performed while the patient was in prone position using a dedicated breast coil. The breast MRI protocol also includes a sagittal T1-weighted localizing sequence followed by a sagittal T2-weighted sequence (3,200/96 [repetition time ms/echo time ms]). A T1-weighted 3-dimensional, fat-suppressed fast spoiled gradient-echo sequence (11.0/4.8; flip angle, 90°; bandwidth, 150 Hz) was then performed before and 4 times sequentially after a rapid bolus intravenous injection of 0.1 mmol/kg body weight of gadolinium at an injection rate of 2.0 ml/s. Image acquisition began immediately after administration of the contrast material and saline bolus.

#### Image analyses

Analysis of PET/CT images was performed by two nuclear medicine physicians and MR-mammography was read by two radiologists. The evaluating physicians were blinded to the results of the other imaging procedures.

#### Supine-PET/CT and mammo-PET/CT

Assessments of the primary tumour, axillary lymph node metastasis, and distant metastasis with PET/CT were based on qualitative and quantitative assessments. PET/CT data were assessed qualitatively for regions of focally increased glucose metabolism and semi-quantitatively by maximal standardized uptake values (SUVmax). A lesion was determined as malignancy or metastasis on PET/CT if FDG uptake was higher than that in the surrounding tissue on qualitative analysis; the SUVmax was checked for the corresponding lesions. We also calculated the SUVmax ratio of primary breast lesion (SUVmax of primary lesion/SUVmax of adjacent muscle) and suspected meta-static lymph node in the axillary fossa (SUVmax of ipsilateral, metastatic axillary lymph node/SUVmax of adjacent muscle).

A breast lesion was suspected as malignant if it showed contrast enhancement, compared with the surrounding tissue (attenuation measurement with regions of interest (ROI) and expressed in Hounsfield unit (HU)) and elevated FDG uptake, compared with the adjacent breast tissue. The volume of ipsilateral axillary fossa (cm^3^) was evaluated for potential lymph node metastasis. Axillary fossa was assessed by measuring the area of axillary fat (between the outer margin of the *latissimusdorsi/major teres* muscle and *minor/major pectoralis* muscle).^24^ Lymph nodes were graded as malignant or benign based on their size and morphologic pattern (cross-sectional short axis diameter of more than 10 mm, loss of fatty hilum or cortical thickening supported the diagnosis of lymph node metastasis). In addition, FDG uptake of axillary lymph node was assessed using qualitative and quantitative analyses.

Supine-PET/CT and mammo-PET/CT were compared for assessment of the primary tumour and axillary lymph nodes. All PET/CT images were reviewed in 3 orthogonal planes (axial, coronal, and sagittal).

#### MR-mammography

Determination of malignancy on MR-mammography was based on assessment of tumour morphological characteristics, as well as the pattern of contrast-enhancement. Breast lesions were rated according to the American College of Radiology Breast Imaging Reporting and Data System lexicon. Both the morphologic appearance (size, shape, and enhancement pattern) and the temporal enhancement pattern were evaluated. Time–signal-intensity curves (progressive, plateau, or washout) were generated for all enhancing lesions.

Evaluation and staging of malignant lesions was performed according to the American Joint Committee on Cancer (AJCC) staging classification. The 2 imaging modalities (PET/CT and MR-mammography) were compared for T stage and focality of the primary tumour. Findings of focality of the primary tumour with mammo-PET/CT and MR-mammography were classified as unifocal (a single lesion in 1 quadrant), multifocal (more than 2 lesions in the same quadrant), or multicentric lesions (more than 2 quadrants affected by breast cancer, or distance between breast cancer lesions more than 4 cm within 1 quadrant).

### Data analyses

Accuracy for delineation of primary tumour and axillary lymph node metastasis among the supine-PET/CT, mammo-PET/CT, and MR-mammography was calculated. T stage of the primary tumour was assessed with supine-PET/CT, mammo-PET/CT, and MR-mammography. Axillary lymph node was assessed between the supine-PET/CT and mammo-PET/CT. In addition, the ability for accurate differentiation of unifocal, multifocal, and multicentric lesions was compared between mammo-PET/CT and MR -mammography. The final diagnosis was served by the histopathologic results.

### Statistical Analyses

Accuracy for assessment of the primary tumour and axillary lymph nodes was calculated according to a patient-based analysis. Differences between images and histopathologic results were evaluated using a paired t-test, Pearson chi-square test, and Fisher’s exact test. A *p*-value of less than 0.05 indicated a significant difference. SPSS version 12.0 (IBM, New York, USA) was used in performance of all statistical analyses.

## Results

### Assessment of the primary tumour

Pathologic biopsy showed that invasive ductal carcinoma, invasive lobular carcinoma and invasive micropapillary carcinoma were found 77.5% (31/40), 5% (2/40) and 5% (2/40) of patients. Ductal carcinoma in situ (DCIS) and the others (small cell carcinoma and tubular carcinoma) were found 7.5% (3/40) and 5% (2/40) of patients.

#### Comparison between supine-PET/CT and mammo-PET/CT

**FIGURE 2.**
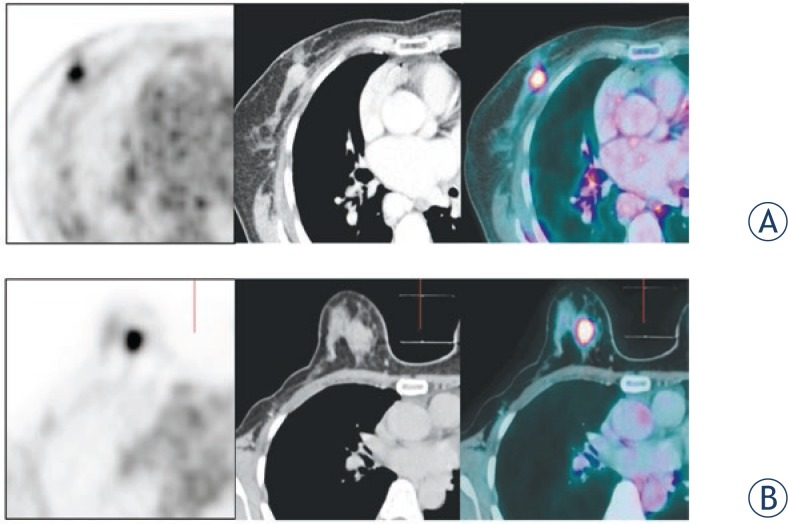
A woman with right breast cancer performed the supine-PET/CT and mammo-PET/CT. A The primary tumour was abutted on the chest wall in the supine-PET/CT. BThe tumour could be more clearly distinguished from the chest wall in the mammo-PET/CT.

The mean size of primary tumour lesions, in the longest axis dimension, was significantly larger on mammo-PET/CT than on supine-PET/CT (supine-PET/CT: 1.3 ± 0.6 cm, mammo-PET/CT: 1.5 ± 0.6 cm, p < 0.001). The difference of tumour size between PET/CT imaging and histopathologic result was 0.5 ± 1.1 cm on supine-PET/CT and 0.2 ± 1.2 cm on mammo-PET/CT. Thus, the real size of the primary tumour was more accurate on mammo-PET/CT than on supine-PET/CT. The mean tumour to chest wall distance (supine-PET/CT: 1.8 ± 0.9 cm, mammo-PET/CT: 2.2 ± 2.1 cm, p < 0.001) and tumour to skin distance (supine-PET/CT: 1.5 ± 0.8 cm, mammo-PET/CT: 2.1 ± 1.4 cm, p < 0.001) were significantly longer on mammo-PET/CT than on supine-PET/CT, indicating better delineation of the tumour from the chest wall and skin (Figure 2, Table 1). The mean SUVmax of the primary tumour was 7.6 ± 5.8 (range: 1.4–20.8) on supine-PET/CT and 8.0 ± 6.2 (range: 1.8–21.4) on mammo-PET/CT (p = 0.02).The mean SUVmax ratio of the primary tumour showed 5.4 (range: 1.4∼30.8) in the supine-PET/CT and 5.7 (1.3∼23.4) in the mammo-PET/CT. No significant difference was showed between the supine-PET/CT and mammo-PET/CT statistically (p = 0.18).

Six patients were proven with T1a stage, and 1 patient with T1b, 21 patients with T1c, 11 patients with T2, and 1 patient with T3 by histopathologic confirmation. None of the patients had T4 stage of breast cancer. Characterization of T stage in breast cancer lesions was corrected in 27 (67.5%) of 40 patients on supine-PET/CT and 29 (72.5%) on mammo-PET/CT, compared with histopathologic confirmation (Table 1). No statistically significant difference was observed (p = 0.63).

#### Comparison between mammo-PET/CT and MR-mammography

In comparison of T stage, mammo-PET/CT provided correct classification of T stage of the primary tumour in 72.5% of cases. In patients with T1a, T1b, and T1c stage, mammo-PET/CT correctly found 2 (33.3%) of 6 patients, 1 (100%) of 1 patient, and 18 (87.5%) of 21 patients, respectively. Mammo-PET/CT also correctly found 8 (72.7%) of 11 patients in the T2 stage and none of the patients had T3 or T4 stage on mammo-PET/CT. MR-mammography provided correct characterization of T stage in a total of 28 (70%) of 40 patients. In patients with T1 (T1a-T1c) stage, MR-mammography found 1 (100%) of 1 patient with T1b, and 19 (90.5%) of 21 patients with T1c. In T2 stage patients, MR-mammography correctly found 8 (72.5%) of 11 patients and none of the patients had T3 or T4 stage in MR-mammography (p = 0.81) (Table 2). In measuring the size of primary tumour, one case showed more correct result in the mammo-PET/CT than the MR-mammography although the T stage was not changed.

In the focality of primary breast lesions, mammo-PET/CT provided correct characterization of 38 (95%) of 40 patients and MR-mammography found 36 (90%) of 40 patients. Solitary, multifocal, and multicentric lesions were found in 33, 2, and 5 patients in histopathologic confirmation. Finally, mammo-PET/CT found 32 of 33 patients with a solitary lesion, 1 of 2 patients with multifocal lesions, and 5 of 5 patients with multicentric lesions. MR-mammography correctly found 31 of 33 patients with a solitary lesion, 1 of 2 patients with multi-focal lesions, and 4 of 5 patients with multicentric lesions (p = 0.68) (Table 3).

#### Axillary lymph node and distant metastasis

In axillary lymph node dissection, intra-axillary lymph node metastasis was histopathologically proven in 18 of 40 patients. PET/CT provided correct detection of intra-axillary metastastic lymph nodes in 14 of 18 patients and the other 4 patients showed no significant FDG uptake and negative imaging findings. No significant differences between supine-PET/CT and mammo-PET/CT were evaluated in the detection of the number of meta-static axillary lymph nodes. Sensitivity, specificity, and accuracy of PET/CT (supine-PET/CT and mammo-PET/CT) for detection of metastatic axillary lymph nodes were 77.8%, 86.4%, and 82.5%, respectively. The mean SUVmax of these meta-static lymph nodes was 4.8 ± 3.9 (range: 1.4 – 13.0) on supine-PET/CT and 5.3 ± 4.4 (range: 2.0 – 14.3). The mean SUVmax ratio of these metastatic lymph nodes showed 1.3 (range: 1.0 – 10.4) in the supine-PET/CT and 1.4 (range: 1.2 – 10.6). There was a no significant difference statistically (p = 0.25).

**FIGURE 3.**
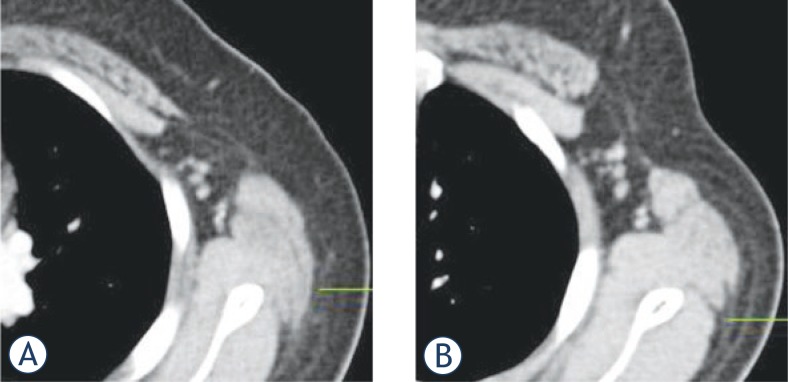
Assessment of axillary fossa in the woman with left breast cancer. A The volume of left axillary fossa measured 15 cm^3^ in the supine-PET/CT. B The volume of axillary fossa measured 24 cm^3^ in the mammo-PET/CT and the assessment of axillary fossa for the potential of metastatic axillary lymph node can be more clear and easy tudy group.

Although no significant differences between supine-PET/CT and mammo-PET/CT were detected in the number of metastatic axillary lymph nodes, the volume of ipsilateral axillary fossa was also significantly wider on mammo-PET/CT than on supine-PET/CT (supine-PET/CT: 21.7 ± 8.7 cm^3^, mammo-PET/CT: 23.4 ± 10.4 cm^3^, p = 0.03) (Figure 3, Table 1).

No distant metastases were found in any of the patients in this study; therefore, PET/CT including mammo-PET/CT did not influence further therapeutic decisions in any patient.

## Discussions

According to the Central Cancer Registry Program 2009, the frequency of breast cancer was 15.1% among women in 2007 and it was reported to be second in cancer occurrence among Korean women, following thyroid cancer.^25^ In Korea, breast cancer has shown a significant increase every year. Therefore, early detection of breast cancer is associated with attainment of cure through early intervention.^26^

Initial breast cancer staging has been based on a multimodality approach: X-ray mammography, ultrasonography, MRI, sentinel lymph node biopsy, and bone scintigraphy. However, this multi-modality approach is time consuming and uncomfortable for patients. Thus, a noninvasive, single-session approach to breast cancer staging may be desirable.^11^–^16^

F-18 FDG PET/CT has been reported to be useful in staging, restaging, and monitoring of treatment response in cancer patients. PET/CT has high sensitivity in detection of the primary tumour and axillary lymph node metastasis, as well as distant metastasis in patients with breast cancer. However, with CT as part of PET/CT, tumour delineation is more difficult to determine than with MRI and PET/CT has been used predominantly for evaluation of distant metastases.^19^–^23^

Because the glandular and fatty tissues of the breast are not uncompressed, prone breast positioning like that used in MR-mammography has a substantial advantage in better differentiation of the primary tumour from its adjacent structures and may improve the assessment of potential infiltration of tumour into the chest wall or skin of the breast. Theoretically, prone positioning may provide more accurate information on the primary tumour and axillary lymph node in PET/CT. In addition, due to enhanced anatomical visualization, the axillary fossa may be evaluated more easily for potential metastatic lymph nodes. MRI has been known to be a sensitive but less specific imaging modality for detection and characterization of breast cancer lesions. Findings from the current study suggest that the protocol for PET/CT-mammography may have similar sensitivity to MR-mammography for detection of intra-mammary lesions.^27^–^29^ However, the diagnostic accuracy of PET/CT-mammography has not yet been defined. We assessed the diagnostic accuracy of PET/CT-mammography as a single diagnostic modality in preoperative staging of breast cancer patients, compared with conventional PET/CT and MR-mammography.

Orel *et al.*^30^ reported that MRI showed high sensitivity (91–100%) for staging of invasive breast cancer, and other studies have also reported that the sensitivity of MRI was 93%; however, specificity was relatively low (65%) due to the lack of ability to characterize enhancing lesions as benign or malignant.^31^–^33^ MRI showed 85% accuracy in breast cancer stage; Heusner *et al.*^34^ reported that MRI showed 77% accuracy for T stage, and 73% accuracy for focality of the primary tumour.

Antoch *et al.*^12^ reported that MRI showed 52% accuracy for T stage and 79% accuracy for N stage; therefore, this result showed lower accuracy than that of another study due to the use of whole body MRI. This study showed that MR-mammography had 70% accuracy for T stage, and 90% for focality of the primary tumour. In comparison with previous studies, MR-mammography showed slightly lower accuracy in T stage and greater accuracy in decision of focality.

In a previous study of FDG PET for staging of breast cancer, Samson *et al.*^35^ reported that FDG PET had 89% sensitivity, and Antoch *et al.*^12^ reported that FDG PET/CT showed 80% accuracy in T stage, and 93% accuracy in N stage. According to this data, conventional PET/CT (supine-PET/CT) showed 67.5% accuracy in T stage and mammo-PET/CT showed 72.5% accuracy in T stage, and 95% accuracy for focality of the primary lesion. Supine-PET/CT and mammo-PET/CT showed similar accuracy (77.8%) for N stage. This study showed slightly lower accuracy than that of current studies in T and N stage of breast cancer patients; however, appropriate comparison between the results would be difficult because only a few studies of mammo-PET/CT in breast cancer staging have been conducted. However, like the preceding study, the current study suggests that this PET/CT protocol for breast cancer may be similar to MRI for detection of intra-mammary cancer lesions. The accuracy of assessment of the primary tumour was also similar between mammo-PET/CT and MR-mammography in this study.

Heusner *et al.* reported that the area of axillary fossa (cm^2^) was significantly wider on mammo-PET/CT than on supine-PET/CT and anatomical structures of the axilla may be more easily differentiated from one another.^24^ Although we assessed the volume of axillary fossa and no significant differences were detected in the number of metastatic axillary lymph nodes, this data also showed that the volume of axillary fossa was wider on mammo-PET/CT than on supine-PET/CT.

None of the patients in this study had distant metastasis; however, Heusner *et al.* reported that PET/CT influenced further therapeutic decisions in 5 patients by detecting the unexpected distant metastasis or synchronous malignancy.^34^

There are some technical limitations in acquisition of whole body mammo-PET/CT. We acquired the mammo-PET/CT image after conventional supine-PET/CT. A whole body mammo-PET/CT protocol, instead of the combined whole body supine-PET/CT with mammo-PET/CT, should be discussed, as this would reduce the examination time. However, patient tolerance may limit this protocol, because the acquisition time can be up to 90 minutes. Use of state-of-the-art multislice PET/CT systems will further reduce the examination times, compared with PET/CT scanners, with fewer detector rows. In this setting, a whole body mammo-PET/CT may be clinically feasible.

Based on our data, mammo-PET/CT improved the accuracy in initial staging of breast cancer and showed that the evaluation of axillary fossa was more clearly and easily, compared with supine-PET/CT. Also mammo-PET/CT indicated the similar accuracy with MR-mammography in T stage and was more correct than MR-mammography for defining focality of lesion. Furthermore, PET/CT can acquire whole body image, so has the advantage for detecting the unexpected, distant metastasis and changing the treatment strategy.

## Figures and Tables

**TABLE 1.** Comparison of conventional whole body PET/CT and PET/CT-mammography in characteristics of primary breast lesions and ipsilateral axillary area

	**Whole Body PET/CT (n = 40)**	**PET/CT-mammography (n = 40)**	**p value**
Tumour size (cm)	1.3 ± 0.6	1.5 ± 0.6	< 0.001
Mean T-C^*^ (cm)	1.8 ± 0.9	2.2 ± 2.1	< 0.001
Mean T-S^†^(cm)	1.5 ± 0.8	2.1 ± 1.4	< 0.001
Axillary area (cm^3^)	21.7 ± 8.7	23.4 ± 10.4	0.03
Compatibility with pathologic T-staging (%)	67.5 (27/40)	72.5 (29/40)	0.63

* Tumour-Chest wall distance;

† Tumour-Skin distance;

n = number of patients

**TABLE 2.** T-stage of resected breast cancer and number of patients correctly staged with PET/CT-mammography and MR-mammography

**T-stage**	**Pathologic Confirmation (n = 40)**	**Staged with PET/CT-mammography (n = 40)**	**Staged with MR-mammography (n = 40)**
T1a	6	2 (33.3%)	-
T1b	1	1 (100%)	1 (100%)
T1c	21	18 (87.5%)	19 (90.5%)
T2	11	8 (72.7%)	8 (72.7%)
T3	1	-	-
T4	0	-	-
Total	40	29 (72.5%)	28 (70%)

**TABLE 3.** Comparison of PET/CT-mammography and MR-mammography in focality of primary breast lesions

**Focality**	**Pathologic Confirmation (n = 40)**	**PET/CT-mammography (n = 40)**	**MR-mammography (n = 40)**
Solitary	33	32	31
Multifocal	2	1	1
Multicentric	5	5	4
Total		38 (95%)	36 (90%)
